# Oligodendrocyte progenitor cells in Alzheimer’s disease: from physiology to pathology

**DOI:** 10.1186/s40035-023-00385-7

**Published:** 2023-11-14

**Authors:** Peibin Zou, Chongyun Wu, Timon Cheng-Yi Liu, Rui Duan, Luodan Yang

**Affiliations:** 1 Laboratory of Exercise and Neurobiology, School of Physical Education and Sports Science, South China Normal University, Guangzhou, 510006 China; 2 Department of Neurology, Louisiana State University Health Sciences Center, 1501 Kings Highway, Shreveport, LA 71103 USA

**Keywords:** Oligodendrocyte progenitor cells, Alzheimer’s disease, Senescence, Microenvironment

## Abstract

Oligodendrocyte progenitor cells (OPCs) play pivotal roles in myelin formation and phagocytosis, communicating with neighboring cells and contributing to the integrity of the blood–brain barrier (BBB). However, under the pathological circumstances of Alzheimer’s disease (AD), the brain’s microenvironment undergoes detrimental changes that significantly impact OPCs and their functions. Starting with OPC functions, we delve into the transformation of OPCs to myelin-producing oligodendrocytes, the intricate signaling interactions with other cells in the central nervous system (CNS), and the fascinating process of phagocytosis, which influences the function of OPCs and affects CNS homeostasis. Moreover, we discuss the essential role of OPCs in BBB formation and highlight the critical contribution of OPCs in forming CNS-protective barriers. In the context of AD, the deterioration of the local microenvironment in the brain is discussed, mainly focusing on neuroinflammation, oxidative stress, and the accumulation of toxic proteins. The detrimental changes disturb the delicate balance in the brain, impacting the regenerative capacity of OPCs and compromising myelin integrity. Under pathological conditions, OPCs experience significant alterations in migration and proliferation, leading to impaired differentiation and a reduced ability to produce mature oligodendrocytes. Moreover, myelin degeneration and formation become increasingly active in AD, contributing to progressive neurodegeneration. Finally, we summarize the current therapeutic approaches targeting OPCs in AD. Strategies to revitalize OPC senescence, modulate signaling pathways to enhance OPC differentiation, and explore other potential therapeutic avenues are promising in alleviating the impact of AD on OPCs and CNS function. In conclusion, this review highlights the indispensable role of OPCs in CNS function and their involvement in the pathogenesis of AD. The intricate interplay between OPCs and the AD brain microenvironment underscores the complexity of neurodegenerative diseases. Insights from studying OPCs under pathological conditions provide a foundation for innovative therapeutic strategies targeting OPCs and fostering neurodegeneration. Future research will advance our understanding and management of neurodegenerative diseases, ultimately offering hope for effective treatments and improved quality of life for those affected by AD and related disorders.

## Background

The cellular components of the mammalian central nervous system (CNS) include neurons and glial cells [1]. In the past, neurons were considered signaling cells, and glia were given an under-appreciated name suggesting that glial cells were merely the glue that held the cells together, keeping the nervous system’s architecture intact [2]. However, increasing evidence has shown the essential role of glial cells in the sophisticated structure and dynamics of neural networks [3, 4], including oligodendrocyte progenitor cells (OPCs), one of the precursor cells of glial cells [5]. OPCs, also known as oligodendrocyte precursor cells, NG2-glia, O2A cells, or polydendrocytes, are abundant in both the white matter and the gray matter of the adult CNS, and named for their essential role as a precursor to oligodendrocytes [6–8].

In the past, molecular mechanistic studies on Alzheimer’s disease (AD) were predominantly centered around amyloid plaques and neurofibrillary tangles (NFTs) [9–11]. The intricate interplay between OPCs and the pathogenesis of neurodegenerative disorders [12–14], particularly AD, has received significant attention in recent research [15–18]. OPCs, a type of glial cells that have long been considered merely precursors to oligodendrocytes, have emerged as a key player in various essential functions within the CNS [19]. In this review, we discuss the multifaceted roles of OPCs, including the crucial roles in myelin formation, cellular signaling within the CNS, phagocytic activity, and blood–brain barrier (BBB) formation and repair. Moreover, we delve into the deteriorating microenvironment in the AD brain, highlighting the alterations of OPC-related events and their potential implications in AD prevention and treatment.

Unveiling the intricate functions of OPCs may open new avenues for understanding the underlying mechanisms of neurodegeneration and identifying potential therapeutic targets. Several critical questions remain unanswered, warranting further investigation and exploration. (1) How does the dynamic interplay between OPCs and their surrounding environment influence OPC differentiation into myelin-forming oligodendrocytes? (2) How does the aging process affect OPC function? (3) Will approaches targeting OPC senescence-related changes provide potential therapeutic strategies for AD and related disorders?

By addressing these complex and unresolved questions, we can gain deeper insights into the intricate roles of OPCs in AD and pave the way for developing novel therapeutic approaches targeting these cells. This review aims to provide a comprehensive overview of the current knowledge regarding OPCs in AD and discuss the potential future directions for AD prevention and treatment from the perspective of OPC changes.

## OPC functions

OPCs, known as CNS resident stem cells, originate from the ventricular zone of the brain and spinal cord, and proliferate and migrate to populate the CNS [20]. Strikingly, OPCs are distributed in the CNS and represent a group of migratory and proliferating adult progenitor cells that can differentiate into oligodendrocytes [19]. OPCs express A2B5, as well as oligodendroglial cell lineage markers Olig1, Olig2, Sox10, GPR17, and Nkx2.2, but they are typically characterized by PDGFR-α and NG2 [19, 21]. The most specific OPC marker is PDGFR-α, a receptor for platelet-derived growth factor (PDGF) A [22–25]. PDGF is the most potent OPC mitogen and survival factor produced by astrocytes, endothelial cells, and neurons [22–26]. The functions of OPCs include involvement in the processes of myelination [27], signal transmission [28] and synaptic pruning [29], and differentiation into other types of glial cells [30].

### Cellular physiology of OPCs: proliferation and differentiation to myelinating oligodendrocytes

The primary function of oligodendrocytes is to produce myelin, the elongated cell membrane that tightly surrounds axons to provide support and insulation [19, 31]. Myelin sheaths provide electrical insulation to axons and allow faster nerve signal transmission [32]. In rodents, the optimal functionality of CNS myelin sheaths is observed when the thickness of the myelin sheath remains stable at a G-ratio (inner diameter/outer diameter) of 0.77 [33]. Deviations from this ratio may potentially lead to initiation and development of neurological disorders [34]. Mature oligodendrocytes can myelinate up to 50 axonal segments, although the actual number may vary depending on the specific regions in the CNS [35].

Initially, OPCs, which are multipotent cells, are present throughout the brain, including in the hippocampus and in all layers of the cortex [36]. During embryonic development, OPCs utilize blood vessels as a “pathway” to migrate in a single-cell fashion toward neurons, forming myelin sheaths [37]. During this process, the signaling of the CXC chemokine receptor CXCR2 plays a crucial role in regulating the number of OPCs and facilitating OPC migration [38, 39]. Myelination during development is an inherent process of CNS maturation guided by genetic programming [40]. After their rapid growth and spread during brain development, the cell division and movement rate of OPCs decreased substantially. However, the OPCs remain among the most actively dividing cell populations in the adult CNS [41].

**Fig. 1:**
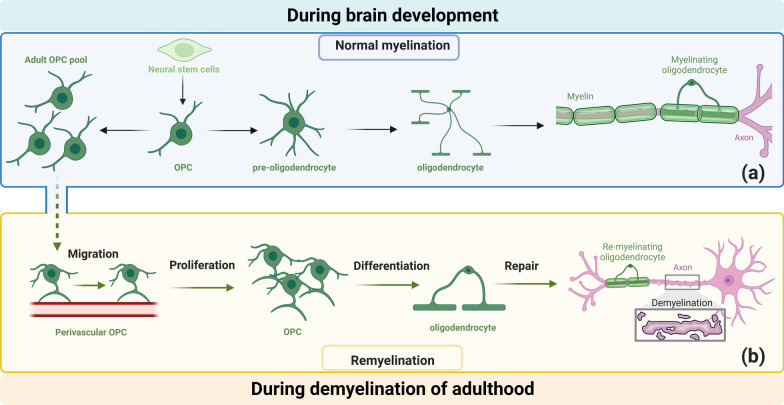
The process of oligodendrocyte progenitor cell (OPC) myelination varies during different stages. **a** During development, OPCs generated from neural stem cells rapidly form myelin sheaths, and a fraction of OPCs will be reserved in the stem cell pool. **b** In adulthood, upon demyelination, OPCs residing in the stem cell pool are swiftly recruited to the site of injury along the vasculature, where they proliferate and differentiate to initiate remyelination

Under appropriate conditions, OPCs receive cues to proliferation and differentiate into oligodendrocytes. As shown in Fig. 1, the OPC differentiation to myelinating cells can be classified into four stages: proliferative OPC stage, pre-oligodendrocyte stage, differentiated oligodendrocyte stage, and myelinating cell stage. The transition between these stages is orchestrated through the intricate interplay of various molecular factors and signaling pathways [42, 43]. As a key step of myelination, OPC differentiation following OPC proliferation and migration are complicated and regulated by a large number of secreted signaling factors that are essential for myelination and remyelination [44–46]. Once OPCs receive differentiation signals, they undergo morphological and molecular changes [47]. The extended processes of OPCs are termed oligodendrocyte processes or lamellipodia, which develop and wrap around axons [19]. The processes gradually compact and form the myelin sheath, a fatty insulating layer surrounding the axon [48].

During myelination, the OPCs undergo a maturation process characterized by expression of specific markers and activation of myelin-related genes [49, 50]. As mentioned previously, the myelin sheath wraps around axons of neurons, resembling the lamellipodia of a ‘liquid croissant’ enveloping the axon [51]. In this configuration, the inner tongue of the lamellipodia wraps around the axon faster than the outer tongue, thereby generating growth polarity and ultimately forming a multi-layered, closed-loop myelin sheath [52]. This process can be initiated by elevated concentrations of phosphatidylinositol-(3,4,5)-triphosphate [52]. The myelination process occurs in spatially and temporally determined sequences [53]. Remarkably, microglia can refine myelin sheaths during development by engaging in phagocytic activity, contributing to the functional modulation of neuronal activity [54].

OPCs are also essential in maintaining the population of oligodendrocytes in remyelination [55]. In response to injury or disease, OPCs differentiate into mature oligodendrocytes to replace damaged or lost myelin [5]. In adulthood, the capacity for migration along blood vessels can be reactivated following myelin damage, allowing OPCs to participate in myelin repair [56]. Remyelination is initiated in response to pathological conditions, including demyelination resulting from injuries, neuroinflammation, or diseases such as multiple sclerosis [57]. Fyn, which belongs to the Src family of kinases, plays a crucial role in facilitating the migration of OPCs by mediating the activation of cyclin-dependent kinase 5 through PDGF signaling. Fyn also contributes to reorganization of the actin cytoskeleton, an essential step in promoting efficient cell migration [58].

Unlike establishing stable and functional myelin during embryonic development, myelin regeneration aims to repair and replace damaged myelin to restore normal impulse conduction. However, the thickness of newly formed myelin sheaths during remyelination is usually thinner than those formed during development, which is one of the characteristics widely used to distinguish between developmental myelination and remyelination [59, 60]. Moreover, the OPCs that are involved in myelination and remyelination are different. The OPCs can be classified into neonatal OPCs (nOPCs) and OPCs that persist into adulthood, named adult OPCs (aOPCs). The expression profile of resting aOPCs is different from that of the nOPCs. However, aOPCs can be activated and revert to a nOPC-like transcriptome. However, the nOPCs generate new oligodendrocytes for myelination, and the aOPCs generate that for remyelination [59, 60].

OPCs possess strict intrinsic proliferation control mechanisms that promote proper cell proliferation and cease cell division while initiating differentiation at the appropriate time [61]. OPC proliferation necessitates the involvement of a diverse range of signaling molecules. Notably, the activation of extracellular signal-regulated protein kinase(s), phosphatidylinositol-3 kinase (PI3K), and p38 mitogen-activated protein kinase is known to trigger the swiftest cellular responses to growth and differentiation factors, as well as a myriad of external stimuli [62–65]. During myelination, the OPCs undergo an extensive and coordinated migration along blood vessels as their physical substrate [66, 67]. During this process, the intrinsic Wnt tone is an essential signal for attracting OPCs to the blood vessels, and the endothelial cells and their products inhibit OPC differentiation during migration [66, 67]. After that, the astrocytes and astrocyte-derived semaphorins 3a and 6a repel the OPCs from the blood vessels, allowing the OPCs to escape from the inhibitory endothelial niche [66, 67]. During the transformation from OPC proliferation to differentiation, several factors are involved in the OPC exit from cell cycle and initiation of differentiation, including the miR-297c-5p targets cyclin T2 (CCNT2), c-Myc, and E2F1 [44–46]. Overall, myelin generation from OPCs to mature oligodendrocytes is a tightly regulated and dynamic process critical for proper functioning of the CNS. Interestingly, it is essential to note that under certain circumstances, OPCs can also give rise to other types of glial cells, which suggests a robust stem cell-like nature of OPCs [6, 68].

### Cellular physiology of OPCs: interaction with other cells in the CNS

OPCs play a role in signal transmission by interaction with other cell types, including neurons and glial cells [66, 69]. The established connections with neurons create an environment for optimal neuronal function [70, 71]. OPCs activate nearby neurons and modulate synaptic transmission by secreting glutamate [72]. The synaptic signaling between neurons and OPCs has been extensively documented in rodent brain regions and human white matter [6, 68, 70, 71]. The synaptic plasticity observed in the neuronal-OPC synapses is similar to that of neuronal-neuronal synapses. Rapid neuron-glial synaptic transmission has been identified between hippocampal neurons and NG2 cells, exhibiting activity-dependent modifications similar as long-term potentiation (LTP) observed in excitatory synapses, a hallmark of neuronal plasticity [73]. However, the distinction sets the neuronal-NG2 synapses apart from numerous other neuronal synapses, as the induction of LTP in these synapses is not accompanied by an increase in the permeability of AMPA receptors to Ca^2+^ [73].

Also, OPCs are critical in maintaining microglial homeostasis within the CNS. Depletion of OPCs leads to microglial activation and exacerbates neuroinflammation [74]. Interestingly, interactions between OPCs and microglia under neuroinflammatory conditions are enhanced [75]. OPCs are capable of producing chemokines that recruit and activate microglia. The interaction between OPCs and microglia is a crucial aspect of the immune response within the CNS [75].

Furthermore, there is an interconnection between OPCs and astrocytes, which has been implicated in brain diseases [76]. For instance, in mice and humans, a neuroglial signaling niche exists between reactive astrocytes and OPCs in white matter stroke [77]. Astrocytes regulate the expression of Sp1R3 on OPCs and promote OPC proliferation through Cx47 signaling in pharmacological analysis [78]. These findings imply that OPCs are “versatile relay stations” for various neuronal signaling pathways.

### Cellular physiology of OPCs: immunomodulatory function

As previously mentioned, the reciprocal interactions regarding neuroinflammation between OPCs and microglial cells suggest that OPCs also play a role in the immune system of the CNS, and some studies have detected the immunomodulatory function of OPCs in CNS diseases [79, 80]. In the experimental autoimmune encephalomyelitis, an animal model of multiple sclerosis (MS), interleukin (IL)-17 induces a cascade of crucial immune responses by interacting with OPCs within the CNS [81]. IL-17 induces a robust pathogenic inflammatory response in OPCs, leading to significantly elevated release of inflammatory cytokines and exacerbating disease progression [81]. Moreover, in a disease context, OPCs exhibit enhanced expression of genes involved in antigen processing and presentation via major histocompatibility complex (MHC)-II, suggesting the immunomodulatory function of OPCs [82]. In addition, the study also found that OPCs have phagocytosis capacity and MHC-II-expressing OPCs contribute to the activation of memory CD4^+^ T cells, suggesting that OPCs may work as an active immunomodulator in MS [82].

However, the CNS environment in demyelination is detrimental for OPCs. The upregulation of interferon-gamma (IFNγ) increases immunoproteasomes and MHC-I molecules, further reducing OPC numbers. These changes cause antigen cross-presentation by OPCs to cytotoxic CD8^+^ T cells, resulting in OPC death [83]. Interestingly, OPCs lacking low-density lipoprotein receptor-related protein 1 (LRP1) in the demyelinated CNS exhibit an anti-inflammatory solid phenotype [80]. The LRP1-deficient OPCs display impaired antigen presentation mechanisms, indicating the inability of the inflammatory response to propagate, thus facilitating faster remyelination and neuroprotection [80]. Furthermore, within the demyelinated environment, OPCs are activated through increased expression of chemokine (C–C motif) ligand 2 and IL-1β to enhance their responsiveness to injury [84]. These changes increase OPC motility and terminal differentiation, contributing to remyelination following demyelinated insults [84]. Together, these observations underscore the essential role of OPCs in immune regulation, neural protection, and the modulation of inflammatory environments.

### Cellular physiology of OPCs: phagocytosis

OPCs are not traditionally considered phagocytic cells, but recent studies have suggested that OPCs are capable of engulfment and pruning under certain conditions [69]. Most of the structural pruning of neurons is believed to be primarily mediated by microglia [85, 86]. However, it has also been found that OPCs possess phagolysosomes and are even more abundant than microglia [69]. In a previous study, high-resolution transmission electron microscopy images showed that the developing mouse cortical OPCs actively participate in axonal pruning [69]. Furthermore, the findings from single-nucleus RNA sequencing demonstrated that OPCs at this stage express crucial phagocytic genes and neuronal transcripts, aligning with active axonal engulfment [69].

Further research revealed that the OPC-mediated synaptic pruning is involved in responses to sensory experience in the developing and adult mouse visual cortex by facilitating thalamocortical synaptic pruning [29]. Therefore, the phagocytic function of OPCs appears to be enhanced by sensory experiences, such as visual deprivation/stimulation paradigms [29]. Interestingly, while participating in shaping neural circuits in the brain, OPCs are also subject to phagocytosis by microglial cells, leading to the formation of intricately refined myelin sheaths and synapses [87]. Notably, NG2 cells cluster around amyloid plaques in APP/PS1 mice and clear β-amyloid peptides through endocytic and autophagic processes in response to the initiation of AD [88]. These findings have opened up new research avenues to better understand the role of OPCs in the brain.

### Cellular physiology of OPCs: contribution to BBB formation and repair

Previous studies suggested that the BBB primarily comprises endothelial cells, glial cells, and pericytes [89]. However, OPCs have been considered as a novel component of the BBB in recent studies [90, 91]. Perivascular OPCs are connected to the brain vasculature endothelial cells through the basement membrane. Therefore, the OPCs are now recognized as an additional cellular constituent of the BBB [91]. OPCs maintain the integrity of BBB by upregulating the expression of tight junction proteins in endothelial cells through TGF-β signaling [92]. During embryonic development, OPCs arise between the perivascular cells and glial cells surrounding the blood vessels, directly participating in the formation of the BBB [91]. Once the BBB is formed, endothelial cells and perivascular cells regulate the proliferation, survival, and differentiation of OPCs by releasing nutrients and signaling molecules [93, 94]. The vascular endothelial growth factor A secreted by endothelial cells regulates the migration of OPCs, which is crucial for the relocation and functions of OPCs [95].

Conversely, OPCs release regulatory factors that modulate the proliferation of perivascular cells and the expression of functionally relevant proteins in endothelial cells [93]. Under normal conditions, most OPCs reside in the parenchymal regions of the adult brain. However, when the BBB is compromised, parenchymal OPCs transform towards perivascular OPCs, significantly increasing perivascular OPCs and the accompanying vascular regeneration [91]. These findings imply that maintaining normal OPC functions is essential during embryonic BBB development, and OPCs play a crucial role in interacting with other vascular cells and spatial migration for the preservation and protection of BBB, and repair of BBB damage.

## AD pathology and harsh microenvironment

AD is the most common neurodegenerative disorder [96]. With the aging of the global population, AD has become a significant burden on the global healthcare system and the leading cause of death among adults older than 65 [97, 98]. AD is characterized by specific neuropathological and biomarker changes, including extracellular accumulation of amyloid-beta (Aβ) peptides and intraneuronal NFTs formed by aggregation of hyperphosphorylated or abnormally-phosphorylated tau proteins [99, 100]. The amyloid hypothesis posits that the Aβ accumulation initiates AD pathogenesis, leading to NFTs, neuronal dysfunction, and dementia [10]. The Tau hypothesis poses that tau hyperphosphorylation induces tau dissociation from the microtubules and aggregation into NFTs, which initiate AD pathology [10]. However, the amyloid and tau hypotheses support that mitochondrial dysfunction, excessive release of neuroinflammatory cytokines, gliosis, and oxidative stress exacerbate AD pathology and induce a harsh microenvironment around cells [10]. The harsh environment affects the cellular physiology of OPCs and neuronal myelination [101].

At the cellular level, the microenvironment can be understood as the extracellular matrix, neighboring cells, bioactive agents such as cytokines, and mechanical forces that collectively influence the functioning of the individual cell [102]. Neuroinflammation, oxidative stress, and mitochondrial damage are significantly elevated in the altered microenvironment of the AD brain [103]. In the presence of multiple pathological features, the microenvironment of the AD brain becomes intricately complex.

Neurotoxicity-related neuroinflammation, oxidative stress, and cytokine release are pathological factors in AD [104, 105]. The accumulation of Aβ and NFTs, along with neuroinflammation and oxidative damage, leads to progressive neurodegeneration [10, 106]. Neuroinflammation is a crucial complex biological process in age-related cerebrovascular and neurodegenerative diseases, such as brain ischemia, AD, and Parkinson’s disease [107]. Studies in AD mouse models have confirmed the significant involvement of neuroinflammatory responses, which induce elevated levels of peripheral inflammatory cytokines and chemokines [108]. AD-related inflammatory components that contribute to neuroinflammation include microglia and astrocytes in the brain, the complement system, and various cytokines and chemokines [109]. The secretion of pro-inflammatory cytokines such as IL-6, IL-1 and tumor necrosis factor-alpha accelerates the progression of inflammation [110]. Interestingly, microglial activation contributes to the clearance of Aβ via phagocytosis and degradation [11]. However, prolonged activation of brain immune cells leads to the release of pro-inflammatory cytokines, initiating an inflammatory cascade and ultimately exacerbating neurodegeneration and neuronal death [111].

Furthermore, oxidative stress serves as an early event in AD and is also regarded as a primary factor contributing to the formation of NFTs in AD [112, 113]. Studies have demonstrated that throughout the disease, the brains of AD patients are exposed to elevated oxidative stress, resulting in lipid oxidation, protein oxidation, a further decline in antioxidant capacity, and increased susceptibility of high-energy-demanding neurons to cell death [112, 114]. Mitochondrial dysfunction drives oxidative stress and inflammation in cells of the brain, shaping the challenging microenvironment in brain injuries and neurodegenerative diseases [115, 116]. Aging neurons exhibit severe mitochondrial fragmentation and cell death, leading to calcium overload and exacerbating microenvironmental deterioration [117].

Moreover, it is crucial to note that mitochondrial dysfunction and structural alterations are prominent features of AD [118]. In AD, there is an aberration in mitochondrial dynamics, shifting from fusion to fission, leading to excessive mitochondrial fragmentation [119]. Additionally, the activities of enzymes related to the oxidative phosphorylation, such as cytochrome *c* oxidase and pyruvate dehydrogenase, are attenuated, indicating decreased ATP production and mitochondrial dysfunction [120]. The structural and functional changes caused by mitochondrial fragmentation lead to release of contents such as mitochondrial DNA (mtDNA) and reactive oxygen species (ROS), which can act as damage-associated molecular patterns (DAMPs) recognized by pattern recognition receptors. The increased DAMPs trigger excessive activation of immune cells in the brain, including astrocytes and microglia, leading to the release of inflammatory cytokines [121].

Traditionally, astrocytes and microglia are the primary immune cells in the CNS [10]. Previous studies have divided the activated astrocytes and microglia into two phenotypes: the neurotoxic pro-inflammatory subtypes A1 (astrocytes) and M1 (microglia), as well as the neuroprotective anti-inflammatory subtypes A2 (astrocytes) and M2 (microglia) [122, 123]. However, single-cell sequencing technology did not detect the existence of classic M1 and M2 phenotypes in vivo [124, 125]. Instead, single-cell analysis has revealed multiple subtypes as well as cellular heterogeneity of microglia and astrocytes [126–128]. Although microglia and astrocytes exhibit changes in transcriptional profile, morphology, and function in diseases, these alterations are similar across various neurodegenerative diseases [128]. Therefore, the activated microglia and astrocytes are called “disease- or degeneration-associated microglia (DAM)” or “disease- or degeneration-associated astrocytes (DAA)” [128]. Like in other neurodegenerative diseases, DAM and DAA have also been identified through single-cell sequencing technology in AD model mice [129, 130]. The pro-inflammatory DAM contribute to the harsh microenvironment by releasing pro-inflammatory cytokines and appear at early AD disease stages, demonstrating better predictive value for pathology even before a cognitive decline occurs [131]. The pro-inflammatory DAM are characterized by expression of pro-inflammatory genes (e.g., Tlr2, Ptgs2, Il12b, and Il1b), while the anti-inflammatory DAM are characterized by phagocytic genes (Igf1, Apoe, and Myo1e) [131]. The DAAs exhibit similar transcriptomics as the DAMs and are observed also before AD-related cognitive decline [129].

A recent study revealed that pathogenic tau proteins can enter microglia, leading to mitochondrial dysfunction and subsequent leakage of mtDNA [132]. This detrimental process exacerbates the microenvironment, further impairing neuronal repair and regeneration [132]. Also, the impairment of the BBB contributes to the onset of the disease [133]. The accumulation of Aβ at sites of vascular leakage leads to inflammatory reactions and cytotoxicity, further exacerbating BBB permeability and accelerating the deterioration of the cellular microenvironment, thus advancing the pathological progression of AD [134]. Therefore, inflammation is not only a passive consequence of the AD process, but also a contributing factor to exacerbating AD pathology [135]. For example, excessive inflammatory cytokine release leads to cellular senescence in various cell types, including OPC senescence [136]. In the process of brain aging in AD, there is an accumulation of iron, which can be attributed to exposure to low levels of H_2_O_2_, leading to worsening of oxidative stress and an impairment of cellular function, further deteriorating the microenvironment of the AD brain [137, 138]. It is worth noting that mechanical force changes also contribute to the deterioration of the microenvironment surrounding OPCs. With age, the “niche” of the brain becomes stiffer, and these mechanical alterations are substantial enough to result in age-related functional decline in OPCs [139]. These findings suggest that the pathological changes in AD are exceedingly complex, wherein the mitochondria-associated changes and excessively activated glial cells form a microenvironment in the brain that fosters a decline in cellular vitality and facilitates aging.

## Alterations in OPC-related events under pathological circumstances of AD

**Fig. 2:**
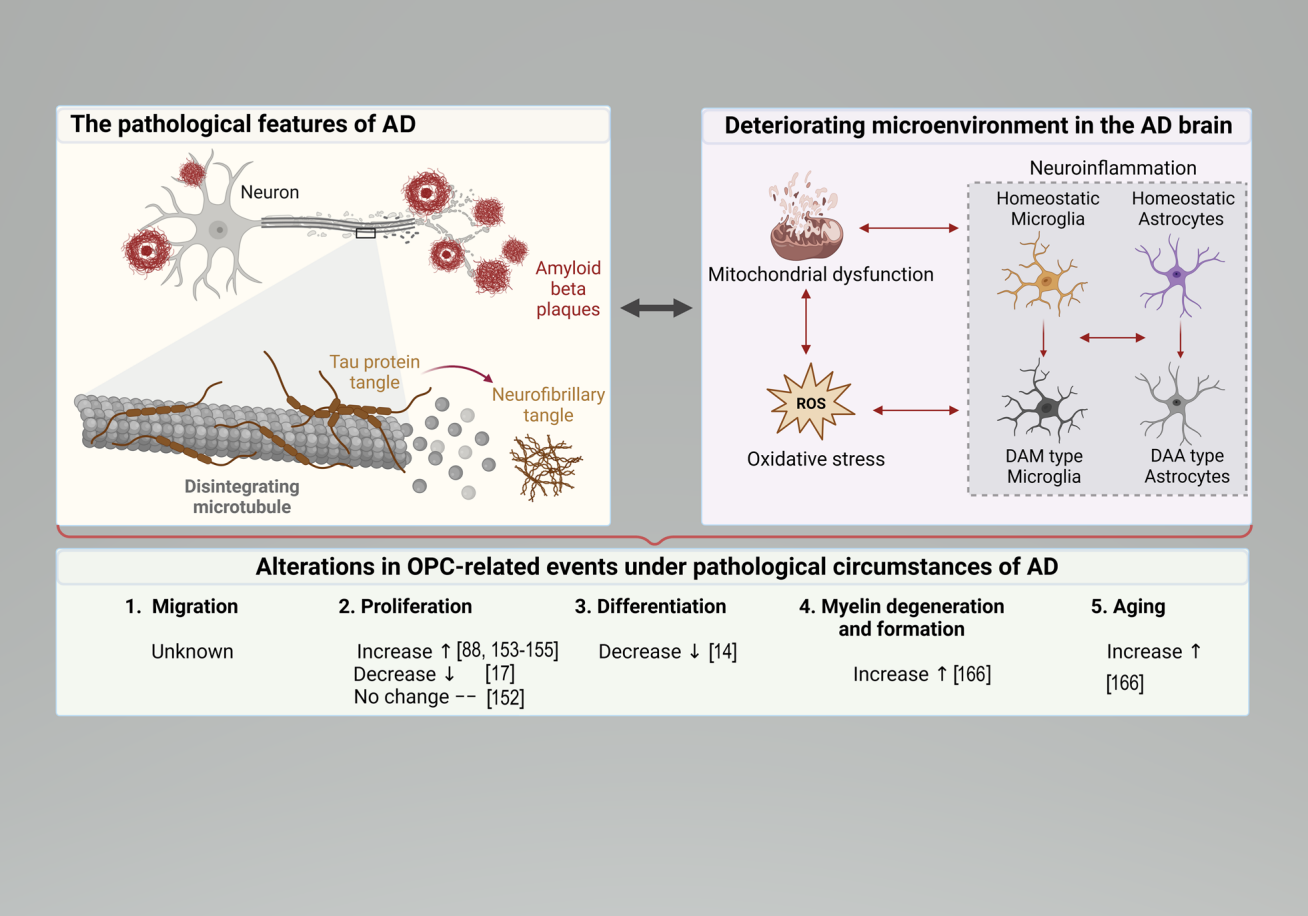
AD pathology and alterations in OPC-related events under pathological circumstances of AD. Under AD pathological conditions, alterations in mitochondrial structure and function result in an overproduction of reactive oxygen species (ROS), leading to oxidative damage of subcellular structures and fragmentation of mitochondria. The oxidative damage and mitochondrial fragmentation further induce inflammatory responses, involving changes in microglial and astrocyte phenotypes. Pro-inflammatory DAM type microglia and DAA type astrocytes release pro-inflammatory cytokines, exacerbating mitochondrial dysfunction and oxidative stress, culminating in a vicious cycle termed “mitochondrial dysfunction-oxidative stress-inflammation response.” Consequently, these changes exacerbate the AD pathology. Within unfavorable cerebral microenvironment, OPC myelination-related events are adversely affected to varying degrees

Both OPCs and oligodendrocytes are closely associated with inflammatory pathology and the toxicity of Aβ, highlighting their involvement in the disease process [140]. Under the harsh microenvironment, as previously mentioned, the biological activity of OPCs is reduced, and the possibility of OPC senescence is significantly increased [141]. The alterations of cellular activity of OPCs under AD pathology primarily result in impaired neuronal axonal health and compromised myelin sheath formation (Fig. 2).

### Changes in OPC migration and proliferation

OPCs migrate to the injured area before undergoing proliferation [47]. Insufficient migration of OPCs to the injured area following demyelination is a determining factor that hinders the timely repair of myelin [142]. Currently, there is a dearth of research on the migratory capacity of OPCs in the AD brain. However, the migration ability of neural stem cells (NSCs), which also possess pluripotency and have the ability to differentiate into OPCs, is compromised due to the senescence of NSCs, suggesting the possibility of impaired OPCs in AD due to cellular senescence [143–145]. Intriguingly, a study on MS, a demyelinating disease that shares similar environments with AD, has found a decline in OPC migration [56, 146]. Interestingly, in the context of MS, OPCs in the vicinity of blood vessels exhibit a phenomenon known as “clustering”, where the OPCs fail to migrate individually along the blood vessels to the demyelinated sites for myelin repair [56]. However, the underlying reasons for OPC clustering around blood vessels and whether a similar phenomenon of blood vessel-associated OPC clustering exists in AD remain unclear [146]. Few studies have investigated OPC migration in the context of AD. Therefore, the impact of OPC migration on AD remains uncertain, necessitating further research to fill the theoretical gap.

Under normal conditions, myelin sheath production relies on the relentless proliferation of OPCs derived from the pool of stem cells [147]. Studies in mice and rats have indicated that aging decreases the OPC proliferation efficiency [139, 148–150]. However, research on monkeys suggests that the efficiency of OPC proliferation remains consistent throughout their lifespan, without any significant decrease observed [151]. In AD pathology, the changes in OPC proliferation become more intricate, with different studies reporting distinct patterns of change (Table 1).

**Table 1:** Changes of OPC proliferation in AD mice

Animal models	Age/months	Sex	Proliferation	Brain area	References
APP/PS1 mice	3, 6, 9, 12, 15, and 18	Male, Female	–	Cortex	[152]
APP/PS1 mice	6, 8	N/A	↑	Cortical gray matter and white matter	[153]
APP/PS1 mice	2	N/A	↑	Hippocampus	[154]
APP/PS1 mice	6	Male	↑	Temporal cortices	[155]
APP/PS1 mice	12, 15	N/A	↑	Cortex	[88]
3×Tg-AD mice	24	Male	↓	Hippocampus	[17]

“↑” represents an increase, “↓” represents a decrease, and “–” represents no observed changes

An early study has indicated that amyloid plaque deposition increases the proliferation of microglia around plaques but does not affect the proliferation of cortical OPCs in double-transgenic AD mice at different ages [152]. Recent investigations, nevertheless, have yielded divergent findings that deviate significantly from the previous study. Between 6 and 9 months of age, there was a notable rise in the population of oligodendrocyte lineage Olig2^+^ cells within the cortical gray matter of APP/PS1 mice, coinciding with the emergence of myelin abnormalities [153]. Furthermore, at as early as 2 months of age, an increase in OPCs was observed in the hippocampus of AD mice, concurrent with a prevalent thickening of myelin [154]. Likewise, an upregulation of NG2^+^ cells was reported in the temporal cortex of the 6-month-old APP/PS1 mice, which coincided with a downregulation of myelin basic protein (MBP) [155]. NG2^+^ cells increase in the cortex and cluster around amyloid plaques at 14 months of age [88].

Furthermore, the NG2^+^ OPCs can internalize and degrade Aβ in culture, supporting their potential involvement in Aβ clearance [88]. These findings suggest that the early proliferation of OPCs in animal models of AD may represent a compensatory response to myelin damage and cytotoxicity of Aβ [156]. However, the increased OPC proliferation does not hold in older AD mice, as the number of OPCs in the hippocampus of 24-month-old 3×Tg-AD mice is significantly lower than that in 6-month-old counterparts [17]. It is evident that with the progression of AD pathology, cellular senescence becomes more pronounced, leading to a significant decline in cell proliferation [18].

The studies mentioned above collectively indicate that the proliferation of OPCs in AD pathology exhibits temporal and spatial variations [17, 18]. Initially, OPC proliferation may be upregulated as a response to counteract the progression of AD pathology. In the later stages, due to the deteriorating brain environment and the declined regenerative capacity [18], the proliferative ability of OPCs diminishes [17, 18].

To summarize, the varying results of the studies mentioned above may largely be attributed to the limited specificity of the antibodies used, with many studies using NG2 or Olig2 as a marker for OPCs. It should be noted that the NG2^+^ cells may potentially represent differentiating OPCs or a subset of cells transitioning into other glial cell types, while Olig2 marks the entire oligodendrocyte lineage [19, 68, 157, 158]. Although further research is warranted to elucidate the impact of AD pathology on OPC proliferation and migration, OPC proliferation and migration could be a potential therapeutic target for AD prevention and treatment.

### Impaired OPC differentiation in AD

OPCs serve as a reservoir of oligodendrocytes, which is crucial for myelin formation and repair [159]. In the pathological context of AD, demyelination is a significant element [160]. The first connection between neurodegeneration in AD and the susceptibility of myelin-forming cells lies in the accumulation of Aβ peptides, which induce dysfunction and death of OPCs and mature oligodendrocytes in vitro and in vivo [161]. Furthermore, emerging scientific investigations have shed light on the interplay between demyelination of neurons and impaired differentiation of OPCs [47]. Due to the prevalent oxidative and carbonyl stress in AD, differentiation of OPCs, which inherently require a high energy supply and are associated with a limited antioxidant capacity, becomes further constrained [14]. These findings imply that efficient differentiation of OPCs is equally important as proliferation in AD.

A study examining the RNA expression profile of post-mortem brain tissues from dementia cases revealed disruption of signaling pathways, contributing to the decline of OPC differentiation [162]. Similar disruptions of signaling pathways involved in OPC differentiation and migration (such as PDGF-2 A and FGF-2) have been observed in MS. These disruptions ultimately lead to a blockade of differentiation during the chronic phase [146, 163]. Additionally, extracellular myelin debris derived from damaged oligodendrocytes inhibits OPC differentiation, and AD is characterized by abundance of such debris [160, 164].

Taken together, under the pathological conditions of AD, the differentiation of OPCs is susceptible to the deterioration of the microenvironment, resulting in a reduction of OPC differentiation capability. Therefore, further research is needed to elucidate the balance between OPC proliferation and differentiation under AD conditions.

### OPC-mediated demyelination and remyelination in AD

Cutting-edge neuroimaging research has greatly advanced our understanding of AD by revealing the presence of demyelination in the white matter. The demyelination process, characterized by a loss of protective myelin sheaths, directly impacts synaptic function as well as learning and memory abilities [160, 165]. In a recent study, a significant loss of pre-existing myelin and increased oligodendrogenesis and remyelination were observed in an AD mouse model, suggesting the existence of both myelin degeneration and remyelination in AD progression [166]. However, the overall levels of myelination were decreased, indicating that although the rate of myelin formation increases in AD, it is difficult to compensate for the increased myelin degeneration rate [166]. Therefore, approaches that could enhance myelination or affect the balance between myelin degeneration and formation may be promising for AD treatment. Further investigations are warranted to explore the balance between myelin degeneration and remyelination under AD.

**Fig. 3:**
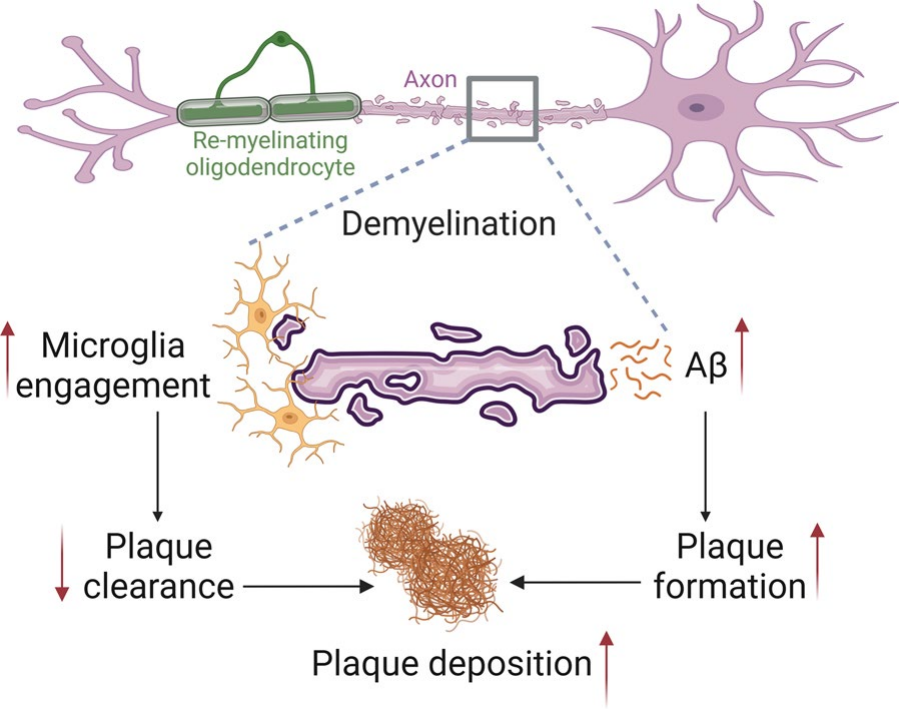
Myelin dysfunction drives Aβ deposition. Myelin dysfunction and demyelination injury are also upstream risk factors contributing to formation of Aβ plaques in AD. Mechanistically, myelin dysfunction may exacerbate the accumulation of the Aβ-producing machinery within axonal swellings and cause the increased cleavage of cortical amyloid precursor protein. In addition, the microglia originally responsible for Aβ clearance are increasingly drawn to demyelination sites, reducing Aβ clearance

Moreover, there is evidence for a correlation between focal demyelination and the presence of Aβ plaques, further emphasizing the link between demyelination and AD [167]. Interestingly, a recent study reported that myelin dysfunction and demyelination injury are also risk factors for Aβ plaque formation in AD (Fig. 3) [168]. Mechanistically, myelin dysfunction may exacerbate the accumulation of the Aβ-producing machinery within axonal swellings and cause increased cleavage of cortical amyloid precursor protein (APP) [168]. In addition, the microglia originally responsible for Aβ clearance are increasingly recruited to demyelination sites, reducing Aβ clearance [168]. Also, recent studies have suggested that the senescence of OPCs plays a significant role in the etiology of demyelination in the contexts of AD and MS [18, 169], suggesting OPC senescence as a potential target for alleviating AD pathology.

### OPC senescence

Cellular senescence refers to a state in which cells are blocked in the G1 phase, unable to proliferate and unresponsive to external stimuli, resulting in the inability to perform normal functions [170]. Cellular senescence is closely associated with the aging of the organism, contributing to cellular functional decline [171]. The senescence-associated secretory phenotype (SASP) is characterized by the extensive release of pro-inflammatory cytokines, cytotoxic mediators, matrix metalloproteinases, and reactive oxygen species. The release of these factors further affects neighboring cells, inducing cellular senescence in the surrounding microenvironment and perpetuating a vicious cycle that accelerates the AD process [172, 173].

Compared to young mice, aged mice exhibit a higher abundance of cellular senescence markers in the brain, indicating increased senescent cells [174, 175]. Previous studies have identified various forms of cellular senescence in neurodegenerative diseases, and these senescent cells have a profound impact on the transmission of signals between neurons and the maintenance of neuronal structures [176, 177].

A human study found that patients diagnosed with late-onset AD had a significantly higher average count of senescent OPCs in the inferior parietal cortex than patients with mild cognitive impairment (MCI) [18]. The number of senescent OPCs in MCI patients was higher compared to the control group without dementia, although the difference was not statistically significant [18]. The results of single-cell sequencing in the frontal cortex indicate a negative correlation between the number of OPCs and amyloid protein [178]. These findings confirmed that patients at a late stage exhibit a decline in white matter integrity and increased cellular senescence compared to the healthy control group [160]. Further investigations on an AD mouse model revealed that Olig2 and NG2 expression in Aβ plaque-associated OPCs exhibited an aging-like phenotype [18]. This change was characterized by the upregulation of p21/CDKN1A and p16/INK4/CDKN2A proteins, and increased activity of the senescence-associated β-galactosidase [18]. The secretion profile associated with cellular senescence, known as SASP, includes a substantial amount of pro-inflammatory cytokines, cytotoxic mediators, metalloproteinases, and reactive oxygen species. These substances have the potential to impact neighboring cells, triggering their senescence and initiating a vicious cycle that accelerates the aging process [173]. According to a previous study, OPCs are required for the maintenance of microglial homeostatic state, and loss of OPCs abolishes the homeostatic microglial state [157], which further promotes the transformation of more microglial cells into the pro-inflammatory type, exerting harmful effects on a more significant number of neurons [179–182]. Therefore, these findings suggest that the loss of OPC function caused by cellular senescence contributes to aging and neurodegenerative diseases. Overall, these studies emphasize the essential role of OPC senescence in forming AD-associated pathology and progression.

### Adaptive myelination in AD

Adaptive myelination, often called activity-dependent myelin plasticity in adulthood, significantly contributes to cognitive function [183]. Experience-driven changes in oligodendrocyte generation are essential for memory consolidation [184], working memory [185], spatial learning [186], and contextual fear memory [187]. These mechanisms are associated with the regulation of OPC proliferation, oligodendrocyte generation, and myelin formation by neuronal activity [188, 189]. However, single-cell sequencing conducted in oligodendrocytes under AD pathology has revealed alterations in genes associated with ion channels and glutamate receptor subunit genes related to neural activity sensing and regulation [190, 191]. Furthermore, adaptive myelination is impaired in a mouse model of chemotherapy-related cognitive impairment. This impairment and associated cognitive deficits can be rescued by the action of a small-molecule TrkB agonist on OPCs [192]. Building upon the previous discussion on myelin loss in AD, it appears that in AD, the adaptive myelination is compromised, potentially linked to weakened signaling between the oligodendrocyte lineage and neuronal activity. However, further research is needed to establish the role of OPCs in this context.

## Current therapeutic approaches targeting OPCs in AD

Over the past few decades, the Aβ and tau protein aggregation hypotheses have been considered the mainstream hypotheses for the pathogenesis of AD and have been extensively targeted as primary therapeutic targets in clinical trials [9, 10]. Unfortunately, to date, nearly all of the interventions targeting Aβ and tau protein hyperphosphorylation in AD have failed in phase III clinical trials [193–195], including immunotherapies, drugs reducing Aβ production or enhancing Aβ clearance, as well as drugs inhibiting tau phosphorylation or aggregation in these years [196–198]. Although Lecanemab, a humanized IgG1 monoclonal antibody targeting Aβ, is approved by the US Food and Drug Administration (FDA) for the treatment of MCI or mild dementia, its efficacy and safety remain controversial [199–202]. Therefore, developing new therapeutic targets for AD is still needed. Interestingly, an increasing body of research indicates that OPCs undergo functional dysregulation in the pathological state of AD, suggesting OPCs as a potential therapeutic target for AD [18, 148, 203]. In the subsequent discussion, we will discuss several existing OPC-based therapeutic approaches for AD (Table 2).

**Table 2:** Current therapeutic approaches targeting OPCs in AD mice

Therapeutic approach	Species	Target	Description	References
Senolytic	APP/PS1 transgenic mice	Senescent OPCs	Utilizes compounds that can selectively clear senescent OPC	[18]
Rejuvenation	Hemizygous hAPP-J20 mice	Senescent OPCs	Mitigates OPC aging by overexpressing anti-aging gene KLOTHO	[209]
Promotion of cell differentiation	3×Tg-AD mice	OPC differentiation-related pathways	Promotes OPC differentiation with RXR agonist	[216]
	APP/PS1 transgenic mice		Promotes OPC differentiation with mTOR inhibitor	[15]

### Senolytic and rejuvenation strategies

In recent years, emerging evidence suggests the therapeutic potential of senescent OPC clearance in AD treatment [18, 176, 204]. Senolytics are a class of compounds that can selectively clear senescent cells by inducing apoptosis of senescent cells [18, 176, 204, 205]. The administration of senolytic treatment effectively targets and eliminates senescent OPCs in the Aβ plaque microenvironment of AD mice [18].

Furthermore, rejuvenation strategies are another widely studied AD therapy [139]. In neurodegenerative diseases, the decline in the functionality of stem cells and progenitor cell populations is a significant cause of reduced tissue regeneration capacity, wherein OPCs are mainly affected [139]. Interestingly, a previous study found that the OPC microenvironment determines OPC senescence and aging [139]. For example, the transfer of aged OPCs into the stem cell pool of young rats significantly improves OPC functionality [139], suggesting that the microenvironment affects cellular senescence. Consistently, another study found that the young cerebrospinal fluid restores oligodendrogenesis and long-term memory consolidation in aged mice via Fgf17 [206]. These studies highlight the importance of mitigating OPC aging for improving AD. Therefore, some studies found that enhancing the expression of the anti-aging gene KLOTHO attenuates amyloid and tau pathology and alleviates cognitive deficits [207–209]. The potential underlying mechanisms include promoting OPC maturation and enhancing remyelination [210–212].

### Strategies targeting OPC differentiation-associated signaling pathways

**Fig. 4:**
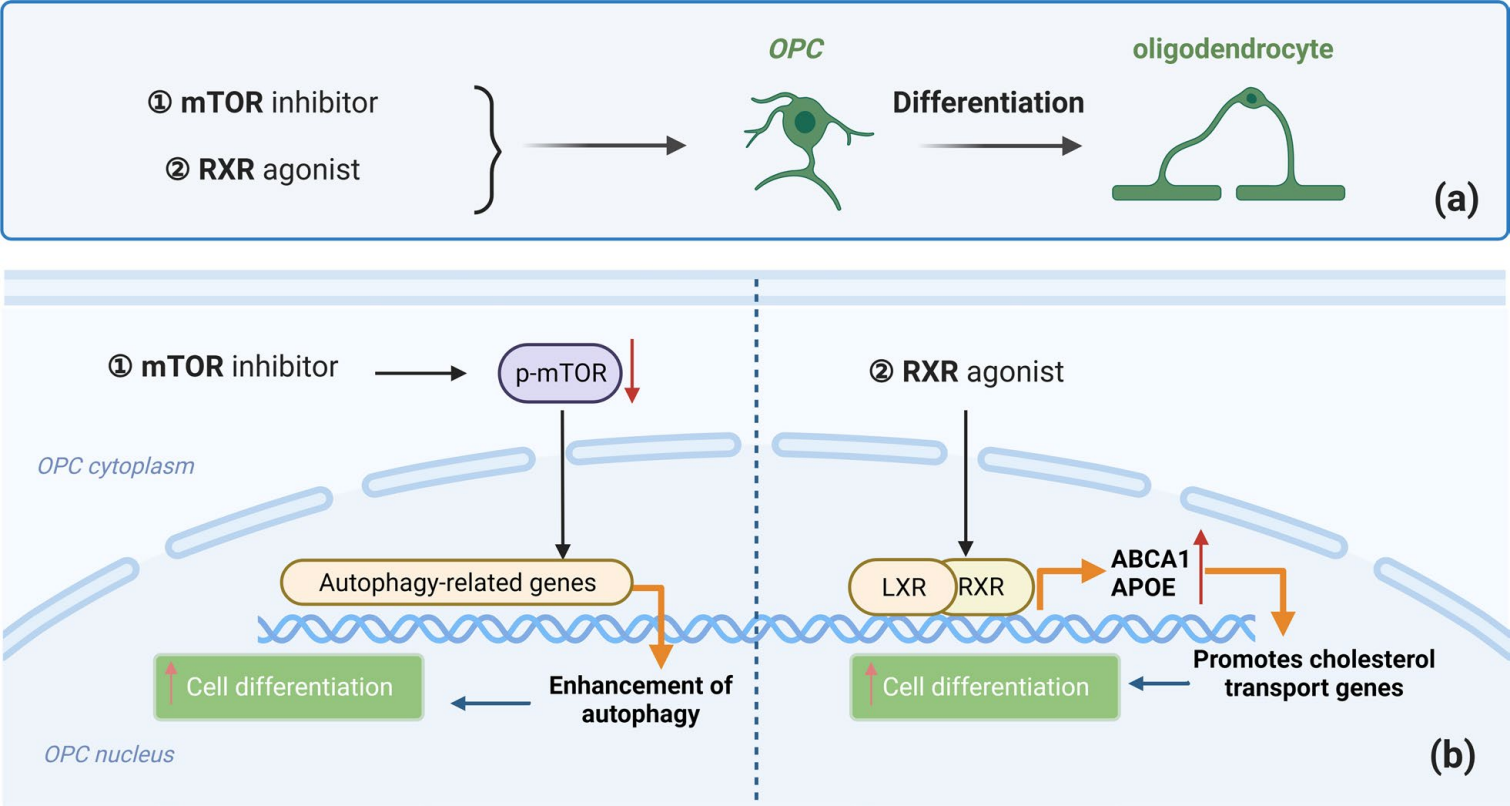
Strategies targeting OPC differentiation-associated signaling pathways in AD. Strategies targeting OPC differentiation-associated signaling pathways include the mammalian target of rapamycin (mTOR) signaling agonist and retinoid X receptor (RXR) signaling agonist. **a** Streamline illustration. **b** Detailed molecular pathway diagrams: The mTOR inhibitor promotes OPC differentiation by downregulating p-mTOR and promoting autophagy-related genes. Elevated autophagy enhances OPC differentiation and myelination. RXR activation promotes Aβ clearance, inhibits Aβ generation, and modulates neuronal function. In addition, the interaction between RXR, ABCA1, and ApoE plays a crucial role in modulating cholesterol transport genes. The enhanced cholesterol transport gene expressions promote OPC differentiation

Previous studies have found that impaired OPC differentiation is the leading cause of remyelination failure in various neurogenerative diseases, including multiple system atrophy, amyotrophic lateral sclerosis, and MS [169, 213–215]. OPC differentiation-promoting therapies exhibit excellent potential in restoring CNS remyelination. For instance, treatment with metformin significantly improves remyelination in aged animals by restoring the regenerative capacity of aged OPCs. Further, metformin promotes the responsiveness of aged OPCs to pro-differentiation signals, suggesting the essential role of OPC differentiation in remyelination [148]. Similarly, several studies have focused on enhancing the signaling pathways involved in OPC differentiation in AD that contains demyelinating environment (Fig. 4). For instance, the signaling pathway of nuclear receptor retinoid X receptor (RXR) upregulates the expression of ATP-binding cassette transporter A1 (ABCA1) and apolipoprotein E (ApoE), thereby directly enhancing the maturation of OPCs and oligodendrocytes, and improving AD-related cognitive function [216, 217]. The interaction between RXR, ABCA1 and ApoE plays a crucial role in modulating lipid metabolism, cholesterol transport, and the pathogenesis of AD [218]. Moreover, RXR activation promotes Aβ clearance, inhibits Aβ generation, modulates neuronal function, and exerts anti-inflammatory actions [219, 220]. RXR agonists activate RXR/LXR and PPAR/RXR heterodimers, promote ABCA1 and ApoE mRNA expression in cells, reduce Aβ levels, and thereby reverse cognitive impairments in AD [221, 222]. Furthermore, RXR agonists enhance the differentiation of OPCs into mature oligodendrocytes, which accelerates CNS remyelination [217]. In addition, RXR activation can also promote remyelination by inducing monocytes, macrophages and microglia to clear myelin debris [223]. RXR serves as a clinically relevant target, and the FDA-approved RXR agonist, bexarotene, has been associated with OPC maturation and remyelination in stroke mice, and cognitive recovery in AD mice [216, 224].

The PI3K/Akt/mammalian target of rapamycin (mTOR) signaling cascade is also an effective target. Donepezil, an FDA-approved acetylcholinesterase inhibitor (AChEI) used in the treatment of AD, has been shown to promote OPC differentiation into oligodendrocytes, enhance myelination, and upregulate myelin-specific proteins [204, 225]. Another compound that acts on OPCs through the mTOR pathway is Clemastine. It is also an FDA-approved antihistamine drug. Clemastine can inhibit OPC aging by downregulating p-mTOR and promoting OPC differentiation, leading to enhancement of autophagy, improved myelin integrity, and reduction in Aβ deposition over long-term treatment, thereby enhancing overall outcomes [15, 166]. Furthermore, the activation of PI3K agonists has been shown to improve peripheral muscle-nerve regeneration [226]. This effect holds promise for alleviating the decline in motor function observed in patients with AD [227]. These studies collectively indicate the significance of targeting and enhancing OPC differentiation signaling pathways in AD therapy.

### Other potential therapeutic approaches

#### Nano-delivery systems

Nano-delivery systems possess finely-tuned physicochemical properties and can be widely used to enhance drug delivery by ameliorating unfavorable drug characteristics, enhancing permeability, and improving tissue distribution and in vivo metabolism [228, 229]. A nanoparticle-based strategy has been developed to enhance the differentiation of OPCs into mature oligodendrocytes capable of repairing myelin. This strategy involves the construction of poly(lactic-co-glycolic acid) nanoparticles with a diameter of approximately 120 nm, which are surface-functionalized with NG-2 antibodies. In a previous study, this nanoparticle was used to deliver leukemia inhibitory factor, a promoter of myelination, to promote OPC differentiation into mature oligodendrocytes and enhance myelin repair [230].

#### Exosomes

Exosomes are small vesicles derived from the cell membrane of various living cells that are released into body fluids and circulate throughout the body [231–233]. Previous research has indicated that exosomes derived from mesenchymal stem cells (MSC-Exo) have shown potentials to enhance neuroregeneration and promote functional recovery in animal models of CNS disease and injury [234–237]. MSC-Exo can cross the BBB in demyelinated mouse models and target neural cells. The MSC-Exo administration significantly increases mature OPCs, elevates the levels of MBP, reduces neuroinflammation by promoting the M2 phenotype of microglial cells while suppressing the M1 phenotype and their associated cytokines, and decreases the level of APP [238]. Currently, a specific exosome targeting OPCs contains a lentivirus with a PDGFRα ligand capable of anchoring to the membrane [239, 240]. This targeted approach towards OPCs in the affected areas significantly enhances the protective capacity of myelin sheaths and promotes OPC differentiation [239, 240].

#### AMPK activators

Several AMPK activators also hold therapeutic potential for AD [241]. For example, disrupted OPC differentiation has been observed in the aging brain [242]. Interventions such as the administration of metformin or dietary restrictions have shown the ability to alleviate the pathological changes of OPCs and restore the regenerative capacity [148]. These changes presumably result from enhanced mitochondrial function and remyelination in aged demyelinated animals [148]. Moreover, as mentioned previously, metformin has been shown to improve myelin recovery in animal models of demyelination induced by cuprizone and alleviate the antioxidant response [243]. Curcumin, another AMPK activator administered by loading into dendrimer nanoparticles, can penetrate the BBB and exert neuroprotective effects by enhancing OPC proliferation and migration [244].

#### Stem cell therapy

Increasing OPCs in patients with AD can represent increased oligodendrocyte production to compensate for the loss of oligodendrocytes in the disease [245]. It has been suggested that stem cell therapy is a direct approach to increasing OPCs [246]. As early as 2005, researchers utilized neural sphere culture to manually select and generate OPCs, which were then transplanted into mice, resulting in increased differentiation into oligodendrocytes and denser myelin sheaths [246]. Subsequent studies have focused on using growth factors such as Sox10, Olig2, and insulin-like growth factor-I for induction [247, 248]. Research on ischemic stroke has revealed that transplantation of OPCs can contribute to the protection of the BBB and the inhibition of brain damage [249]. However, the distribution of OPCs is complex, and when considering transplantation, the regional specificity and the effects require serious consideration [68].

#### Exercise

As a “non-invasive” intervention, physical exercise has gained widespread recognition for its role in preventing and treating various diseases [250, 251]. While the effects of exercise on OPCs do not target a specific pathway, it is cost-effective and provides multiple benefits [106, 252]. In addition, innovative motor tasks, such as the skilled reaching task or complex running wheel, have been found to stimulate the proliferation, migration, and differentiation of OPCs in rodents, leading to oligodendrocyte generation and remyelination [253–256]. In the brains of AD patients, the activities of antioxidant enzymes SOD2 and CAT are significantly reduced [257]. However, long-term exercise can dramatically increase the contents of SOD2 and CAT, enhancing antioxidative capacity [106, 257]. Exercise can regulate the release of irisin from muscles, which regulates the release of brain-derived neurotrophic factors and promotes the ability of synaptic mitochondria to transport glucose and enhance respiratory coupling efficiency. Furthermore, exercise regulates mitochondrial regeneration by increasing the expression of PGC-1α, thus influencing the number of mitochondria [258]. More importantly, exercise is essential in maintaining the dynamic balance of mitochondrial fission and fusion and the structural integrity of mitochondria [259]. Therefore, exercise regulation on OPCs may be achieved by improving the brain microenvironment [260].

#### Photobiomodulation (PBM)

Similarly, a growing body of research has demonstrated that PBM by applying low-level laser (light) exerts beneficial effects in various neurodegenerative disorders [261–263]. In animal models of demyelinating diseases, PBM has been shown to stimulate OPC proliferation and promote myelin repair, thereby enhancing neuroplasticity and facilitating disease recovery [264, 265]. A recent study revealed that in a rat model of early-life adversity, there is a decrease of OPC proliferation and differentiation, an increase of myelin loss, and elevated levels of oxidative damage. However, these changes can be reversed through early PBM treatment [266]. Unfortunately, no reported evidence regarding PBM therapy has been shown to specifically target OPCs in AD. However, it is worth noting that studies have investigated the involvement of myelin sheath repair in AD [11, 267]. In particular, research in transgenic AD rodent models has shown promising results regarding the effects of PBM therapy on MBP^+^ myelin-related changes [11]. PBM has been widely studied to enhance the activity of mitochondrial cytochrome *c* oxidase, reduce oxidative damage, and improve antioxidant enzyme activity [10]. Therefore, mitochondrial protection, in turn, can improve the microenvironment associated with AD [11, 268, 269]. These studies suggest that PBM may promote the recovery of various brain disorders by regulating OPC differentiation and myelin formation. However, additional research is still needed to investigate the precise targets of PBM.

## Conclusion and future perspectives

In conclusion, OPCs play critical roles in the CNS, contributing to myelination, intercellular signaling, phagocytosis, and BBB formation and repair [47, 79]. However, in the context of AD, the microenvironment in the brain deteriorates, leading to alterations in OPC-related events [137–139]. Under pathological conditions, the impaired migration and proliferation of OPCs compromise the differentiation of OPCs into mature oligodendrocytes [47]. The disrupted OPC differentiation contributes to myelin degeneration and pathological change in neurodegenerative diseases.

Additionally, OPC senescence contributes to demyelination in AD [176]. Given the significance of OPCs in AD, current therapeutic approaches target OPC senescence and function. Strategies focusing on improving OPC senescence, rejuvenating OPC functions, enhancing myelin repair processes, promoting OPC differentiation, facilitating remyelination, and restoring neuronal circuitry exhibit great potential for AD treatment. Moreover, there are other potential therapeutic approaches beyond OPC targeting. Combining strategies targeting OPCs or microenvironments may provide synergistic effects and enhance therapeutic outcomes. Therefore, further research is warranted to deepen our understanding of the intricate mechanisms underlying OPC-related events in AD pathology. Unraveling the specific signaling pathways, molecular players, and cellular interactions involved will pave the way for more precise and effective therapeutic interventions. Additionally, exploring the potential of emerging technologies, such as gene editing, holds promise in harnessing the regenerative capacity of OPCs and promoting myelin repair in AD [270]. In addition, considering the association between AD and the pathological loss of functional synapses [271–273], it is crucial to ascertain the potential role of OPCs in synaptic phagocytosis, which may underlie the neurobiological deficits observed in AD and related disorders.

In summary, elucidating the complex interplay between OPC, the microenvironment, and AD pathology is crucial for developing targeted therapeutic strategies. Focusing on rejuvenating senescent OPCs, modulating differentiation pathways, and addressing the altered microenvironment may help mitigate myelin abnormalities and restore cognitive function in AD patients.

## Data Availability

Not applicable.

## Abbreviations

OPC: Oligodendrocyte progenitor cell
nOPC: Neonatal oligodendrocyte progenitor cell
aOPC: Adult oligodendrocyte progenitor cell
BBB: Blood–brain barrier
AD: Alzheimer’s disease
CNS: Central nervous system
IFNγ: Interferon-gamma
IL: Interleukin
MHC: Major histocompatibility complex
LRP1: Low-density lipoprotein receptor-related protein 1
DAM: Disease-associated microglia
DAA: Disease-associated astrocyte
PI3K: Phosphatidylinositol-3 kinase
LTP: Long-term potentiation
Aβ: Amyloid-beta
NFT: Neurofibrillary tangle
mtDNA: Mitochondrial DNA
DAMP: Damage-associated molecular pattern
SASP: Senescence-associated secretory phenotype
MCI: Mild cognitive impairment
RXR: Receptor retinoid X receptor
ABCA1: ATP-binding cassette transporter A1
ApoE: Apolipoprotein E
APP: Amyloid precursor protein
PBM: Photobiomodulation
